# Ivermectin‐Creme 10 mg/g allein oder in Kombination mit oralem Doxycyclin bei Patienten mit perioraler Dermatitis (POD): eine retrospektive Fallserie

**DOI:** 10.1111/ddg.15691_g

**Published:** 2025-06-11

**Authors:** Martin Schaller, Daniela Lenders, Gabi Handgretinger, Andrea Gawaz

**Affiliations:** ^1^ Universitätshautklinik Tübingen, Tübingen

Sehr geehrte Herausgeber,

Die periorale Dermatitis (POD), auch rosazeaähnliche Dermatitis genannt, ist eine relativ häufige entzündliche, chronische Hauterkrankung des Gesichts.^1^, ^2^, ^3^ Sie ist durch stechende, brennende, juckende und oft beidseitige erythematöse Papulovesikel oder Papulopusteln gekennzeichnet, die über Wochen bis Monate bestehen bleiben können. Obwohl der periorale Bereich am häufigsten betroffen ist, können die Hautveränderungen auch periorbital und paranasal auftreten. Die meisten Patienten (ca. 90 % der Fälle) sind Frauen im Alter zwischen 20 und 45 Jahren.^4^, ^5^

Die Ätiologie der POD ist nach wie vor ungeklärt. Man vermutet jedoch, dass sie multifaktoriell bedingt ist. Die Rolle topischer Steroide als Auslöser der POD ist gut belegt. Darüber hinaus werden übertriebene Hautpflege, insbesondere die langfristige Anwendung von Gesichtscremes, die zur Beeinträchtigung der epidermalen Barrierefunktion führt, sowie die Anwendung fluoridhaltiger Zahnpasta als mögliche Ursachen der POD vermutet.^1^, ^3^

Eine pathogenetische Rolle wird auch für mikrobielle Faktoren wie Infektionen mit *Fusobacterium* spp., *Candida albicans* und *Demodex folliculorum* diskutiert. Dies stimmt mit der kürzlich veröffentlichten Beobachtung überein, dass Anti‐COVID‐19‐Gesichtsmasken ein weiterer möglicher prädisponierender Faktor sein könnten, da sie über eine Erhöhung der Gesichtstemperatur eine Veränderung des Hautmikrobioms (Proliferation von *Staphylococcus epidermidis* und/oder *Fusobacterium* spp. und/oder *Demodex folliculorum*) und der Durchlässigkeit der Hautbarriere bewirken.^6^ Darüber hinaus fanden Dolenc‐Voljc et al.^7^ eine signifikant höhere Milbenprävalenz und Milbendichte bei Patienten mit POD, die zuvor mit topischen Steroiden behandelt worden waren im Vergleich zu POD‐Patienten ohne Steroid‐Vorbehandlung und zu gesunden Kontrollpersonen (für beide p < 0,001).

Bislang beruht die Behandlung der POD hauptsächlich auf klinischen Erfahrungswerten, es gibt nur wenige gut kontrollierte Therapiestudien.^2^ Zu den Therapieoptionen gehören das Absetzen und strikte Vermeidung aller topischen Steroide, Kosmetika und Salben (Nulltherapie), antientzündliche und antimikrobielle Externa mit Erythromycin, Metronidazol, Azelainsäure oder Pimecrolimus‐Creme und systemische Antibiotika (Minocyclin, Tetracyclin, Doxycyclin) sowie in schweren Fällen orale Retinoide.^1^, ^2^

In Anbetracht der potenziellen Rolle von *Demodex folliculorum* in der Pathogenese der POD wie bei der Rosazea erscheint die Behandlung mit topischem Ivermectin, einem makrozyklischen Lakton‐Disaccharid‐Antiparasitikum, sinnvoll. Ivermectin 10 mg/g Creme (Soolantra^®^, Galderma Laboratorium GmbH) einmal täglich aufgetragen ist in den USA und in Europa seit 2014 beziehungsweise 2015 zur Behandlung entzündlicher Läsionen (Papeln, Pusteln) bei Rosazea im Erwachsenenalter zugelassen. Der genaue Mechanismus, der für die Wirksamkeit des Medikaments bei Rosazea verantwortlich ist, ist noch nicht geklärt; es wird jedoch angenommen, dass die therapeutische Wirkung vor allem auf seiner entzündungshemmenden und antiparasitären Wirkung beruht.^8^, ^9^, ^10^ Aufgrund dieser Eigenschaften schien es vielversprechend, POD‐Patienten mit vorheriger topischer Steroidtherapie (hohe Demodexdichte) und ohne vorherige Steroidtherapie (normale Demodexdichte) mit Ivermectin 10 mg/g Creme zu behandeln. Die Entscheidung über die jeweilige Therapie wurde vom behandelnden Arzt getroffen. Die Studie und die Datenerhebung erfolgten mit Zustimmung der Ethikkommission der Universität Tübingen (Projektnummer 856/2023BO2).

Bislang gibt es nur eine Fallserie mit acht Patienten, die einen Therapieerfolg bei der Behandlung von POD mit topischem Ivermectin gezeigt hat.^9^ Diese vielversprechenden Ergebnisse veranlassten uns, eine retrospektive Fallserie mit fünf POD‐Patienten (21–68 Jahre) durchzuführen, die eine *Off‐Label*‐Behandlung mit Ivermectin 10 mg/g Creme einmal täglich als Monotherapie oder in Kombination mit Doxycyclin 40 mg Kapseln mit modifizierter Wirkstofffreisetzung erhielten. Die Dauer der Erkrankung reichte von einem bis zu mehreren Monaten, und bis auf einen Patienten waren alle mit verschiedenen topischen Wirkstoffen vorbehandelt worden (Tabelle 1).

**TABELLE 1:** Demographische Daten der Patienten und Behandlungserfolg mit Ivermectin 10 mg/g Creme als Monotherapie oder in Kombination mit Doxycyclin 40 mg mit modifizierter Freisetzung.

Pat. Nr.	Geschlecht	Alter (Jahre)	Bestandsdauer der Symptome	Vortherapie(n)	Demodex‐Milben/ *Standardized skin surface biopsy* (SSSB)	Aktuelle Therapie	Anwendung	Verlauf
1	Weiblich	47	1 Monat	Topisches Steroid (Clobetasolpropionat)	> 5 Demodex‐Milben/cm^2^ (14 Milben in 2 Proben)	Topisches Ivermectin 1 %	0‐0‐1	Gute Verbesserung nach 8 Wochen, vollständige Abheilung nach 16 Wochen
2	Weiblich	58	6 Monate	Topisch: Steroid, Erythromycin, Clotrimazol	> 15 Demodex‐Milben/cm^2^ (22 Milben in 2 Proben)	Topisches Ivermectin 1 %	0‐0‐1	Gute Verbesserung nach 8 Wochen
3	Weiblich	21	6 Monate	Keine	Nicht durchgeführt	Topisches Ivermectin 1 % + Doxycyclin 40 mg	0‐0‐1 0‐0‐1	Ausgezeichnete Verbesserung/vollständige Abheilung nach 8 Wochen
4	Weiblich	62	Mehrere Monate	Topisch Dimetinden maleat, topisches Steroid	Kein Nachweis von Demodex‐Milben (3 x)	Topisches Ivermectin 1 % + Doxycyclin 40 mg	0‐0‐1 0‐0‐1	Keine Verbesserung mit topischem Ivermectin nach 3 Wochen; nach Hinzunahme von Doxycyclin ausgezeichnete Verbesserung nach 6 Wochen, vollständige Abheilung nach 13 Wochen
5	Weiblich	68	Unbekannt	Topisches Steroid, antibiotische Salbe	Nicht durchgeführt	Topisches Ivermectin 1 % + Doxycyclin 40 mg	0‐0‐1 0‐0‐1	Ausgezeichnete Verbesserung/vollständige Abheilung nach 6 Wochen

***Hinweis***: Der Behandlungserfolg wurde anhand der *Investigator‐Global‐Assessment‐*Skala (IGA) bewertet, mit IGA 0 – hervorragende Verbesserung, IGA 1–2 – gute Verbesserung, IGA 3–4 – geringe/keine Verbesserung

Den Patienten wurde geraten, sämtliche Externa, einschließlich topischer Glukokortikoide, Kosmetika und Salben, abzusetzen. Alle sprachen sehr gut auf die Behandlung mit topischem Ivermectin an. Das Ergebnis wurde anhand der *Investigator‐Global‐Assessment* (IGA)‐Skala evaluiert (IGA 0 – ausgezeichnete Verbesserung, IGA 1–2 – gute Verbesserung, IGA 3–4 – geringe/keine Verbesserung). Zwei Patienten erreichten eine gute Verbesserung (IGA 1–2) nach 8 Wochen mit topischem Ivermectin allein (Abbildung 1), zwei Patienten eine ausgezeichnete Verbesserung/vollständige Remission (IGA 0) mit der anfänglichen Kombination aus topischem Ivermectin 10 mg/g Creme einmal täglich und Doxycyclin 40 mg nach 6 beziehungsweise 8 Wochen (Abbildung 2), und ein Patient nach der Zugabe von Doxycyclin eine ausgezeichnete Verbesserung (IGA 0) nach 6 Wochen und eine vollständige Remission nach 13 Wochen.

**ABBILDUNG 1:**
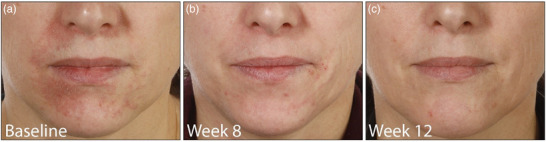
Patient (a) vor, (b) nach 8 Wochen und (c) nach 12 Wochen topischer Behandlung mit Ivermectin 10 mg/g Creme einmal täglich als Monotherapie mit guter Verbesserung.

**ABBILDUNG 2:**
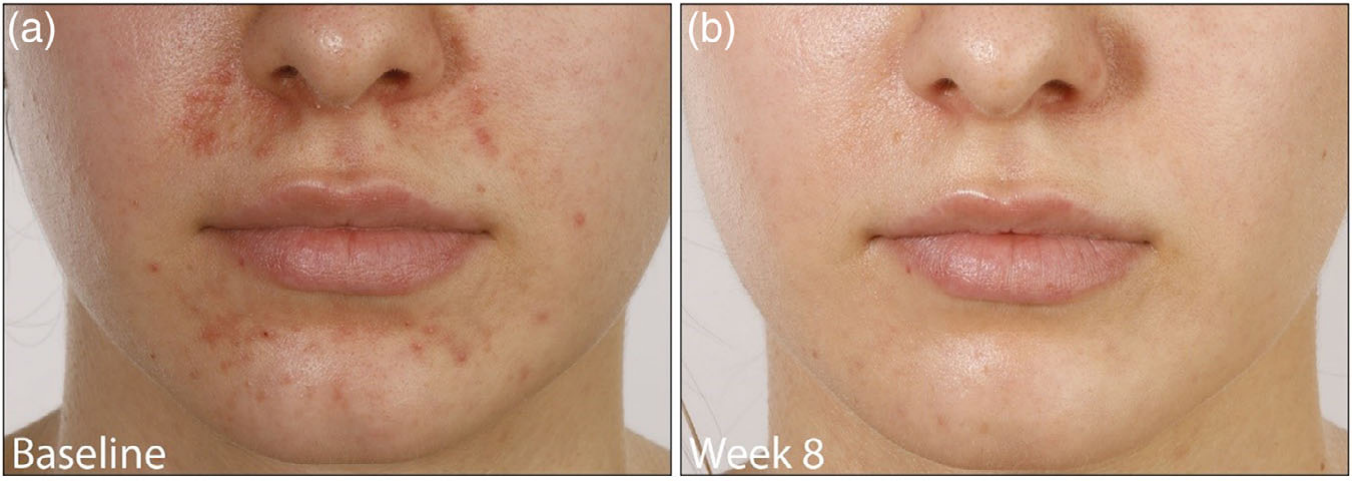
Patient (a) vor und (b) nach 8 Wochen topischer Behandlung mit Ivermectin 10 mg/g Creme einmal täglich in Kombination mit Doxycyclin 40 mg mit modifizierter Freisetzung mit ausgezeichneter Verbesserung/vollständiger Remission.

Zusammenfassend zeigte sich, dass topisches Ivermectin 10 mg/g allein oder in Kombination mit oralem Doxycyclin 40 mg für die Behandlung von POD bei Patienten im Alter von 21–68 Jahren gut wirksam und verträglich war. Insbesondere die Kombination von Ivermectin 10 mg/g Creme mit Doxycyclin 40 mg zeigte eine ausgezeichnete Heilungsrate und gute Wirksamkeit bei steroid‐ und nichtsteroid‐induzierten Formen der POD. Unsere Ergebnisse bestätigen, dass topisches Ivermectin eine mögliche Option bei erwachsenen Patienten mit POD ist, die weitere Studien rechtfertigt.

Einschränkungen dieser Studie ergeben sich aus der geringen Zahl der behandelten Patienten und dem retrospektiven Studiendesign. Zur Bestätigung der Ergebnisse sind groß angelegte prospektive Studien erforderlich.

## DANKSAGUNG

Assistenz beim Schreiben erhielten wir von Frau Dr. Katrina Recker, finanziert von Galderma Laboratorium GmbH.

Open access Veröffentlichung ermöglicht und organisiert durch Projekt DEAL.

## INTERESSENKONFLIKT

M.S. übte in den vergangenen drei Jahren Beratertätigkeiten für Abbvie, Bayer, Galderma, L'Oréal, und UCB aus und erhielt Vortragshonorare von AbbVie, Galderma, Janssen‐Cilag, Lilly, Mibe, Med Update, und Omnicuris. D.L., G.H. und A.G. erklären keine Interessenskonflikte.
